# A Single-Walled Carbon Nanotube Network Gas Sensing Device

**DOI:** 10.3390/s110807763

**Published:** 2011-08-08

**Authors:** Li-Chun Wang, Kea-Tiong Tang, I-Ju Teng, Cheng-Tzu Kuo, Cheng-Long Ho, Han-Wen Kuo, Tseng-Hsiung Su, Shang-Ren Yang, Gia-Nan Shi, Chang-Ping Chang

**Affiliations:** 1 Department of Materials Science and Engineering, National Chiao Tung University, Hsinchu 30013, Taiwan; E-Mails: rubsico@ms28.hinet.net (L.-C.W.); eru7023@gmail.com (I-J.T.); ctkuo@mail.nctu.edu.tw (C.-T.K.); 2 Department of Electrical Engineering, National Tsing Hua University, No. 101, Sec. 2, Kuang-Fu Road, Hsinchu 30013, Taiwan; E-Mail: upper@seed.net.tw (S.-R.Y.); 3 Analytical Chemistry Section, Chung-Shan Institute of Science & Technology, Hsinchu 30325, Taiwan; E-Mails: johnnie.ho1213@msa.hinet.net (C.-L.H.); herman.h.kuo@gmail.com (H.-W.K.); pandasu@gate.sinica.edu.tw (T.-H.S.); 4 Department of Applied Chemistry & Materials Science, Chung Cheng Institute of Technology, National Defense University, Taoyuan 33448, Taiwan; E-Mails: gianan.shi@msa.hinet.net (G.-N.S.); cpchang1@ndu.edu.tw (C.-P.C.)

**Keywords:** single-walled carbon nanotube (SWCNT) networks, gas sensing device, chemical vapors

## Abstract

The goal of this research was to develop a chemical gas sensing device based on single-walled carbon nanotube (SWCNT) networks. The SWCNT networks are synthesized on Al_2_O_3_-deposted SiO_2_/Si substrates with 10 nm-thick Fe as the catalyst precursor layer using microwave plasma chemical vapor deposition (MPCVD). The development of interconnected SWCNT networks can be exploited to recognize the identities of different chemical gases by the strength of their particular surface adsorptive and desorptive responses to various types of chemical vapors. The physical responses on the surface of the SWCNT networks cause superficial changes in the electric charge that can be converted into electronic signals for identification. In this study, we tested NO_2_ and NH_3_ vapors at ppm levels at room temperature with our self-made gas sensing device, which was able to obtain responses to sensitivity changes with a concentration of 10 ppm for NO_2_ and 24 ppm for NH_3_.

## 1. Introduction

Carbon nanotubes (CNTs) are molecular scale quantum wires exhibiting many unique properties for potential nano-devices applications [1–6]. One of the applications is as the sensing materials in a gas sensor device. Single-walled carbon nanotubes (SWCNTs), multi-walled CNTs (MWCNTs) and randomly oriented nanotube networks for detecting chemical gases and vapors have been a subject of active research [7–10]. Some devices were designed to detect the changes in the resistance of the Schottky barriers among the nanotubes and their metal contacts [11], which could be used to improve the real-time sensing to monitor the combustible gases, gas leakage and environmental pollution. However, a few studies have shown the main disadvantages of this kind of gas sensor, which include operating at a high temperature, requiring higher conductivities, and needing a clean sensing chamber.

In this study, we demonstrate the simple and rapid manipulation of carbon nanotube materials using direct growth manipulation to build a practical nano-device. These CNT-assisted sensor devices also showed excellent sensitivity and a rapid response time to detect NO_2_ and NH_3_ with the aim of obtaining the sensor’s detection limit by observing the changes in electric resistance.

## 2. Experiment

### 2.1. Growth of SWCNT Networks

The SWCNT networks used in this work were grown by a MPCVD system. The synthesis process has been described elsewhere in detail [12,13]. In brief, an Al_2_O_3_ layer of 10 nm was first deposited on SiO_2_/Si wafer substrates, followed by an iron (Fe) film with a thickness of 10 nm. The SWCNT networks were grown on the substrates using a microwave plasma at a power of 750 W in a chamber pressure of 12 Torr under constant gas flows of H_2_ (400 sccm) and CH_4_ (1.5 sccm) for 10 min. The substrate temperature was estimated to be ∼650 °C during the growth of the SWCNT networks. Prior to the deposition process, the Fe/Al_2_O_3_-coated samples were pretreated by 30 Torr hydrogen plasma under the condition of a 100 sccm H_2_ gas flow rate with an input microwave power of 600 W for 15 min. The grown products were characterized by field emission scanning electron microscopy (FESEM, JEOL-6500), high resolution transmission electron microscopy (HRTEM, JEM-2100F), and micro-Raman spectroscopy (Renishaw RM-1000, excitation laser: 514.5 nm in wavelength, laser spot: ∼5 μm in diameter).

### 2.2. Gas Sensing Setup and Measurement

**Figure 1.**
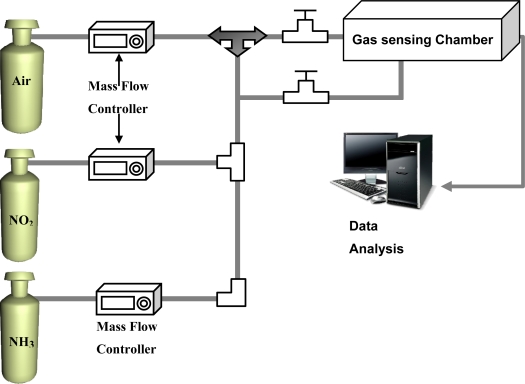
The experimental setup used in the gas sensing.

The gas sensing devices were prepared by directly soldering the two electrical leads in an air atmosphere on the surface of the SWCNT networks deposited on the SiO_2_/Si substrates. The distance between the two electrodes was 1 cm. Figure 1 shows the experimental setup used in the measurements. For safety, the experiments were conducted under a hood. The sensor device was placed inside the gas sensing chamber with 0.1 L capacity. Dry air was used as background gas and the flow rate was set at 100 sccm. The concentration of NO_2_ and NH_3_ gas were controlled by mass flow controller. For gas sensing, the reaction chamber was alternately purged by the dried-and-filtered air and testing gas, respectively, with a duration time of 30 s for each under a flow rate of 100 sccm. The resistance between the two electrodes on the SWCNT networks as a function of purging time was recorded every second by a computer interfaced with an electrical resistance meter through a GPIB bus.

## 3. Experimental Results and Discussion

### 3.1. Surface Morphology and Microstructure of SWCNT Networks as Gas Sensing Materials

**Figure 2.**
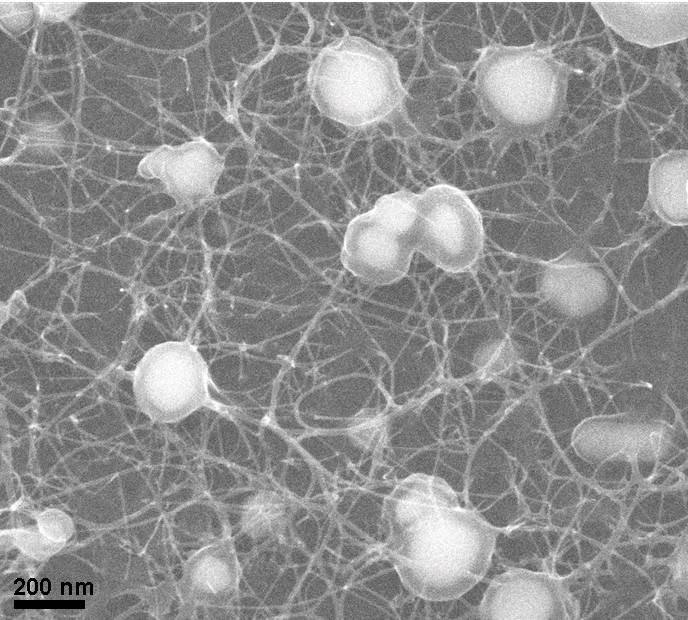
SEM micrograph of the SWCNT networks for gas sensing.

**Figure 3.**
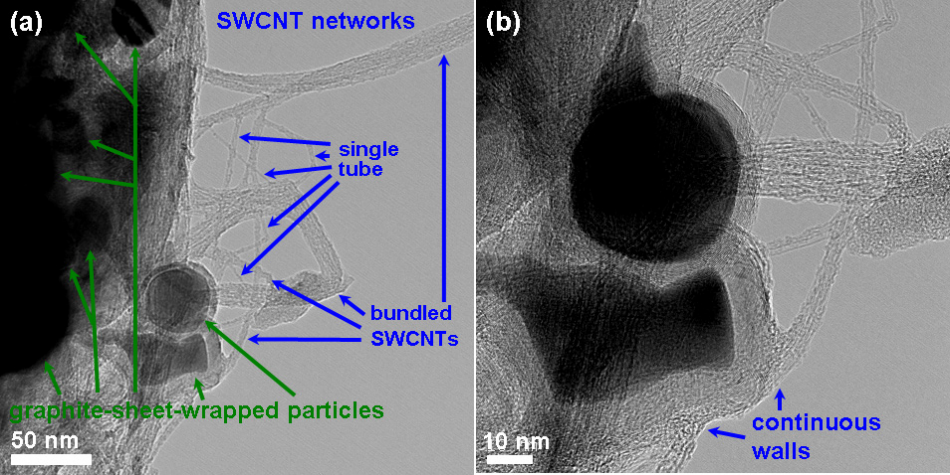
TEM images of the SWCNTs grown from catalyst particles and/or branched from growing tubes.

**Figure 4.**
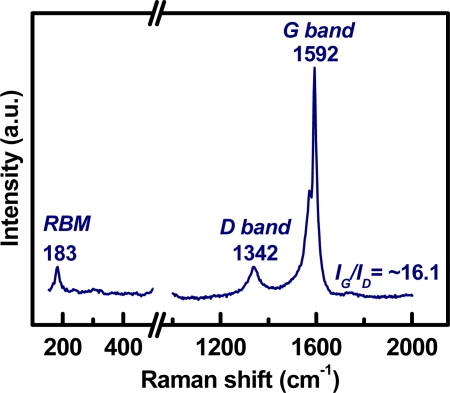
Raman spectrum of the as-grown SWCNTs networks.

**Figure 5.**
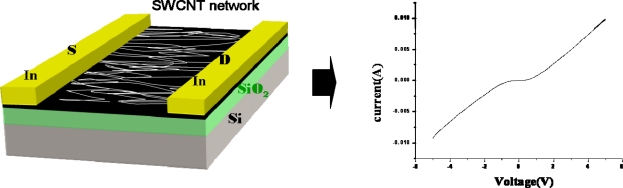
Schematic diagram and I-V curve of the gas sensing device.

Figure 2 shows the SEM micrograph of the as-grown CNT networks on an Al_2_O_3_-deposited SiO_2_/Si substrate. It can be observed that the CNTs are surface-grown and connected to each other between catalyst particles, forming a laterally interconnected network. To examine the structure of the CNTs, the developed CNT networks are further characterized by HRTEM after being ultrasonically dispersed on a carbon grid. A low magnification HRTEM image, as shown in Figure 3(a), reveals that the CNT-based lateral architecture comprises a mixture of graphene-sheet-wrapped catalyst particles and interconnected SWCNTs, alone or assembled into bundles. Moreover, the HRTEM image shown in Figure 3(b) at high magnification further shows that the walls of the SWCNTs connect continuously with the outer layer of the graphitic shells of the given particles. Moreover, to evaluate the structural properties of the prepared SWCNT networks, we also employed Raman scattering, which is one of the most widely used and powerful techniques to characterize CNT samples. Figure 4 shows the Raman spectrum characteristics of our SWCNT networks. The existence of highly-graphitized SWCNTs can be confirmed by a high fitted-peak area ratio of the G-band to the D-band, namely I_G_/I_D_, and the radial breathing modes (RBM) peaks in the low frequency region of 100–300 cm^−1^. The strong signal at about 1,592 cm^−1^ is attributed to the G-band of the tangential mode of a graphite-like material while the peak around 1,342 cm^−1^ is the D-band representing defects in the graphite structures. It is clear that the I_G_/I_D_ ratio is approximately 16.1 for our sample, indicating the growth of highly crystallized SWCNTs in the networks. In addition, the strong RBM peak at around 183 cm^−1^ implies that the majority of tubes are about 1.35 nm in diameter in the resonant case when using the 514.5 nm line of the Ar laser for excitation [14–16]. Figure 5 provides a schematic diagram and I-V curve of the device. The source (S) and drain (D) Indium electrodes were added to this substrate with SWCNTs networks by using low temperature bonding techniques. This makes the process easier for mass production, comparing to the existing CNTFET that needs semiconductor post-fabrication process for electrode making and device packaging.

### 3.2. Gas Sensing Performance

**Figure 6.**
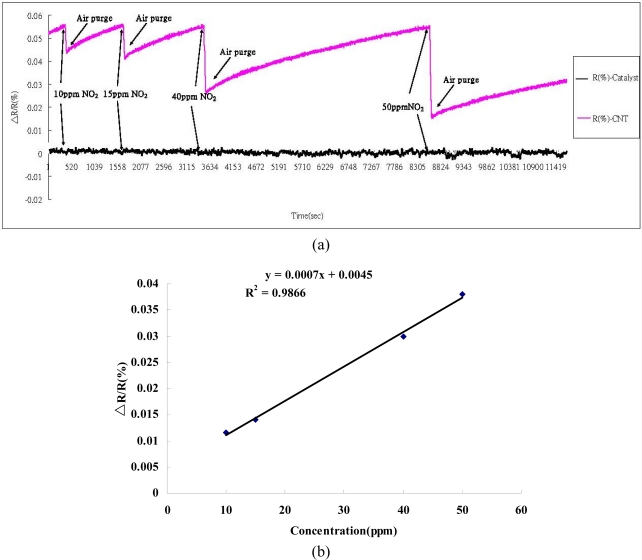
(**a**) The gas sensing devices with SWCNT networks (initial resistances: 44.343 kΩ) and with only Fe catalyst on substrate responding to the NO_2_ gas of 10, 15, 40, and 50 ppm at room temperature, with a gate voltage set at zero, exhibited an increase in response ΔR/R (%) upon exposure to NO_2_; (**b**) The calibration curve was derived from Figure 6(a).

**Figure 7.**
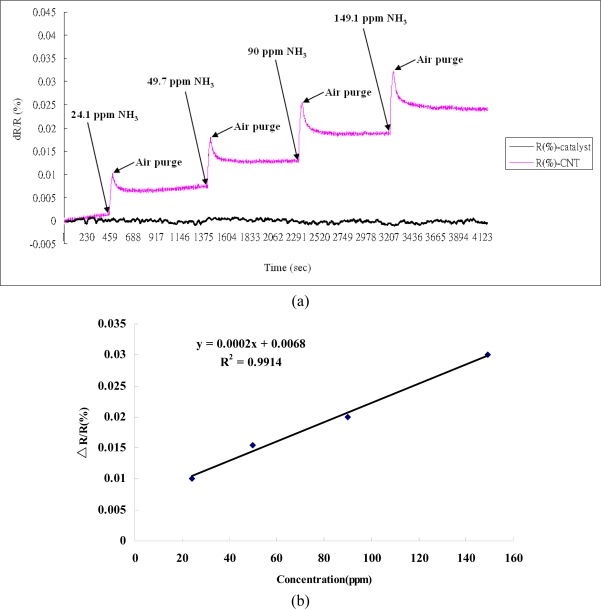
(**a**) The gas sensing devices with SWCNT networks (initial resistances: 65.372 kΩ) and with only Fe catalyst on substrate responding to the NH_3_ gas of 24.1, 49.7, 90, and 149.1 ppm at room temperature, with a gate voltage set at zero, exhibited an increase in response ΔR/R (%) upon exposure to NH_3_; (**b**) The calibration curve was derived from Figure 7(a).

To investigate the responses of the SWCNT networks to gaseous toxic chemicals by measuring the changes in resistance, NO_2_ and NH_3_ gases were used in the experiments. It was obvious that the value of the resistance dropped upon binding of the NO_2_ or NH_3_ molecules [17–20]. This could result in the transfer of electrons from CNTs to NO_2_ molecules, which are strong oxidants bearing an unpaired electron, and thus could lead to a decrease in the resistance of the CNTs when NO_2_ is bound to the surface of semiconducting CNTs (Figure 6). The response to NO_2_ isn't clear because the recovery time is very long. The high bonding energy between SWCNTs and NO_2_ results in our gas sensing devices not reaching saturation during the designed analyte gas exposure time (30 s). This long recovery time has also been reported for carbon nanotube sensors by Kong *et al*. [17]. However, the binding of the NH_3_ gas was observed to cause an elevation in resistance. This may be attributable to the absorption of reductive NH_3_ molecules to the surface of carbon nanotubes, leading to electrons being transferred from NH_3_ molecules to the CNTs’ valence bands (Figure 7) [17,21]. Based on the obtained data, it appears that the influence of gas adsorption on the SWCNTs varied with different gas species, and the self-developed SWCNT networks possessed outstanding sensitivity with semiconducting traits [22,23].

In addition, the larger the superficial measurement is, the more test gas is needed. In the experiment, we concluded that test gases at low ppm levels could lead to low responses from the sensing device. To obtain better responses from the SWCNT networks with a low ppm or even ppb levels, the sensing device would need to be reduced in size [24]. In the test of NH_3_, we found the sensors behaved well when compared to other results [18,20]. There is a constant drift due to an irreversible gas sensing process, which might be due to chemisorption between the defect along the sidewall and the NH_3_ molecules, as reported by Robinson *et al*. [25].

It is well known that both semiconducting and metallic tubes are products of all SWCNTs synthesis methods. The metallic SWCNTs may be burned off after fabrication [26], but this additional processing step makes it more difficult for control and mass production. Bao’s results [27,28] showed that semiconducting and metallic SWCNTs could be separated by using different functionalized surfaces and produces TFT device sensor presented very good device characteristics. The TFT sensors fabricated with aligned, sorted nanotube networks (enriched with semiconductor SWCNTs) showed a higher sensitivity to analytes than those fabricated with random, unsorted networks with predominantly metallic charge transport.

Our gas sensing device fabrication process directly grows the SWCNT networks on the surface of device without using any chemical solution. As a result, the sensing materials on the gas sensing device should be a combination of both semiconducting and metallic tubes. It behaves like a chemiresister sensor. These SWCNTs chemiresistor sensors operate by a change in conductance due to a relatively stronger charge transfer interaction, as opposed to weaker dipole or van der Waals interactions. Consequently, our gas sensing device does not need an additional process to burn off the metallic SWCNTs after fabrication, and functions as a chemiresistor.

These SWCNTs chemiresistor sensors operate by a change in conductance due to a relatively stronger charge transfer interaction, as opposed to weaker dipole or Van der Waals interactions. Carbon nanotubes provide a large surface area for gases passing through such that the sensitivity of gas sensing is greatly enhanced. Therefore, carbon nanotubes are proven to be highly sensitive for detection of low concentration gas molecules. The main detection principle is the charge exchange between SWCNT sidewalls and gas molecules absorbed by the surface of carbon nanotubes. The molecular interactions with defect sites in SWCNT sidewalls strongly affect their electrical response. In our experiments, NO_2_ are electron withdrawing molecules, while NH_3_ are electron donating molecules.

## 4. Conclusions and Future Work

We have demonstrated the use of a gas-sensing material based on SWCNT networks and investigated the influence of various concentrations of NH_3_ and NO_2_ gases within the SWCNT networks in terms of the performance in detecting NH_3_ and NO_2_. The proposed gas sensing device does not need an additional processing step to burn off the metallic SWCNTs after fabrication. This makes the process easier to implement for mass production. In addition, the proposed sensing device functions as a chemiresistor. Source (S) and drain (D) indium electrodes were added to this substrate with SWCNTs networks by using low temperature bonding techniques. This again makes the process more practical for mass production, compared to the existing CNTFET that needs a semiconductor post-fabrication process for electrode making and device packaging. The main improvement was to modify the nanomaterial to increase the compatibility between the two components; therefore, the SWCNT networks can be easily fabricated in the gas sensing material. These would also help us to realize the gas sensing performance of the SWCNT networks materials without using any chemical solution on it.
